# The contribution of cultural identity to subjective well-being in collectivist countries: a study in the context of contemporary Chinese culture

**DOI:** 10.3389/fpsyg.2023.1170669

**Published:** 2023-07-25

**Authors:** Song Zhou, Gaoyu Liu, Yingming Huang, Tingyu Huang, Shiya Lin, Jie Lan, Huaqi Yang, Rongmao Lin

**Affiliations:** School of Psychology, Fujian Normal University, Fuzhou, China

**Keywords:** cultural identity, subjective well-being, collectivism, perspective-taking, Chinese culture

## Abstract

**Introduction:**

Though the important effect of cultural identity on subjective well-being is widely acknowledged, the details of how different cultures’ unique features influence well-being remain to be revealed. To address this issue in the context of Chinese culture, the present study investigates whether and how the prominent features of Chinese culture—collectivism and red culture—shape Chinese people’s subjective well-being.

**Methods:**

The Red Cultural Identity Scale, Subjective Well-Being Scale, Collectivism Scale, and Perspective-Taking Scale were used to assess 1,045 Chinese residents.

**Results:**

The results showed that red cultural identity positively predicted participants’ subjective well-being through the mediated role of collectivism. Furthermore, perspective-taking was found to moderate the mediating effect of collectivism.

**Discussion:**

These results demonstrate that the way cultural identity predicts subjective well-being is highly correlated to specific cultural features, e.g., the opinion of values, which was significant in practice with a cross-cultural background.

## Introduction

Happiness is the eternal pursuit of human beings. Kim-Prieto et al. (2005) defined happiness widely, including positive life satisfaction, quality of life, and subjective evaluation of life in terms of emotion and cognition. Subjective well-being, which refers to the overall evaluation of the assessor’s quality of life according to self-set standards, is considered an important comprehensive psychological index to measure individuals’ quality of life (Diener, 1984). According to the general cultural theory of subjective well-being, culture can be the main factor in constructing the concept of happiness and shaping individuals’ attitudes toward it. Culture not only has a direct effect on subjective well-being but also affects it by shaping self-concept (Lu et al., 2001). However, due to the various cultures around the world, the question remains as to how the unique features of different cultures influence subjective well-being.

It has been suggested that culture amounts to a complex whole including knowledge, belief, art, morals, law, customs, and any other capabilities and habits that can be acquired as a member of society (Johnson, 2013). In the context of modern Chinese culture, red culture occupies an important place. Red culture is referred to as systematic ideological ideas and beliefs formed by the Chinese Communist Party and left-wingers during the Chinese Revolutionary War and further developed by the construction of socialist China. Red culture not only advocates positive optimism and enterprising spirit and cultivates noble sentiment and an optimistic attitude toward life but also shows the pursuit and yearning for the good life and involves strong patriotism (Li, 2020).

Cultural identity, which represents the extent to which one identifies oneself as belonging to a specific culture, is considered a special case of social identity, encompassing ethnic identity that includes feelings toward and behaviors exploring the ethnic group to which one claims heritage (Douglass and Umana-Taylor, 2015). Furthermore, cultural identity also contributes to one’s overall sense of self and belonging (Ferguson et al., 2017). Previous studies on Chinese Canadians and Chinese Americans have shown that cultural identity has a positive effect on people’s subjective well-being through clarity of self-identity, which proves the significance of an individual’s cultural identity to well-being (Usborne and Taylor, 2010).

In summary, there may be a positive correlation between cultural identity and subjective well-being. In contemporary China, identity in red culture represents the recognition of the ideals and values of Chinese revolutionary culture and national cultural identity in social identity and helps shape individuals’ overall sense of self and belonging. If, as general cultural theory argues, the particularity of Chinese red culture has an effect on subjective well-being, the question of how it works is raised.

The present study intends to explore whether and how red cultural identity promotes Chinese residents’ subjective well-being, providing further evidence for research in related fields.

### The mediating effect of collectivism

Collectivism is an important dimension in determining value differences (Hamamura et al., 2013; Hofstede, 2001). It reflects the level of concern an individual has for other individuals and groups (Hui, 1988; Felfe et al., 2008) and represents a strong sense of identity with organizational norms and responsibilities (Triandis, 1996). Chinese red culture, which is derived from socialist ideology, comprises the spirit of selfless dedication (Li, 2020) and the morality of serving society wholeheartedly (Xu, 2011). Indeed, collectivism has been constantly emphasized by the authorities since China’s revolution led by the CCP. Therefore, collectivism can be considered one of the crucial components of red culture. According to the Social Identity Theory, the understanding and construction of the corresponding social identity are prerequisites for being a member of a group (Brown, 2000). It is reasonable that the stronger one’s identification with the red culture, the stronger one’s sense of collectivism.

According to several studies conducted in collectivist countries, such as Spain and India, collectivists frequently experience higher levels of subjective well-being (Rego and Cunha, 2009; Ahuja et al., 2021). People in collectivist societies are more concerned with the well-being of groups, develop better relationships with family and friends (Martella and Maass, 2000), and engage in more altruistic behavior (Brewer, 1999), which result in higher subjective well-being. In terms of cooperation, people with higher levels of collectivism tend to have positive attitudes toward group members (Brewer, 1999), show camaraderie when working in an organizational environment (Rego and Cunha, 2009), and manifest a greater willingness to cooperate with others and help each other (Gouldner, 1960; Deckop et al., 2003; Westwood et al., 2004). As a result, experiences of positive emotions like gratitude, joy, and comfort gained from collectivist activities push individuals toward a greater sense of happiness (Rego and Cunha, 2009). The positive effect of happiness, in turn, causes a significant enhancement in cooperation and trust. This may be due to the enhanced sociability, interpersonal warmth, group involvement, and interpersonal trust caused by positive emotions (Tov and Diener, 2009).

Overall, red cultural identity may promote people’s level of collectivism, which in turn may affect their subjective well-being.

### The moderating role of perspective-taking

Perspective-taking, referring to the social cognitive ability to take the perspective of others and imagine or speculate on their thoughts or attitudes, is the foundation of communicating with others and social interactions (Linde and Labov, 1975; Galinsky et al., 2005; Brown-Schmidt et al., 2008). It typically involves the comprehension of connections between various situations and the emotions they evoke (Cutting and Dunn, 1999). Individuals with higher perspective-taking abilities may be more likely to experience happiness. As Rueda et al. (2014) found in their experiments, there is a significant positive correlation between perspective-taking and happiness. Research by Shanafelt et al. (2005) has also revealed a correlation between perspective-taking and well-being; that is, high psychological well-being is associated with enhanced individual perspective-taking.

Studies have shown that there are important links between perspective-taking and cultural features. For example, Chinese people who live in collectivistic cultures are found to be better at perspective-taking than Americans who live in individualistic cultures (Wu and Keysar, 2007). According to related studies, social identity affects how well perspective-taking works and has an interaction effect on out-group favorability ratings (Tarrant et al., 2012). In a study involving participants from 63 countries, Chopik et al. (2017) discovered that participants from individualistic countries performed worse in perspective-taking tasks compared to those from collectivistic countries. These results demonstrate that individualism increases the prominence of people’s own views in the perspective-taking process, which leads to an individual’s greater egocentric bias in reasoning about others’ mental states. Accordingly, collectivism, as a core feature of red culture, might be moderated by perspective-taking.

As argued by the Affect Value Theory, culture plays an important role in shaping the emotional sensations that people appreciate and would ideally want to experience. Those with a higher level of perspective-taking are more likely to have a higher level of cultural identity, and vice versa (Tsai et al., 2006). Nelson and Baumgarte (2004) proposed that perceived cultural dissimilarity can reduce perspective-taking, whereas perceived cultural similarity can significantly predict perspective-taking (Heinke and Louis, 2009). However, it remains to be explored whether the effect of perspective-taking acts on the relationship between red culture identity and subjective well-being.

The correlation between red cultural identity and subjective well-being may be higher in people with a higher level of perspective-taking, in line with the previous statement. In other words, for individuals with a higher level of perspective-taking, the stronger the red cultural identity, the higher the subjective well-being.

### The current study

**Figure 1:**
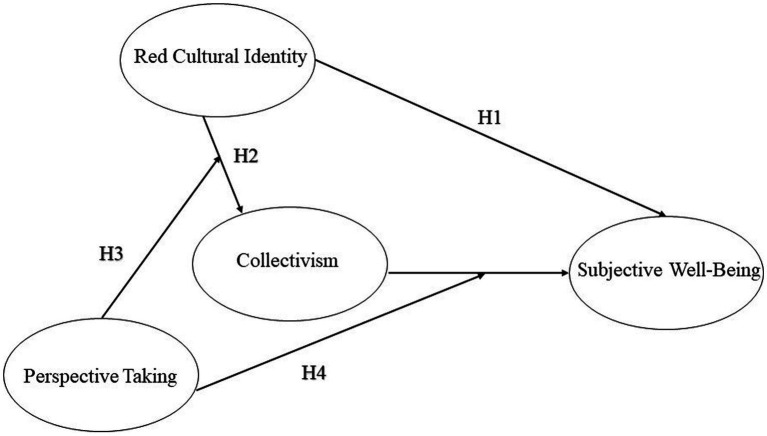
The hypothesized model.

Although there have been suggestions that red cultural identity and subjective well-being are related up to this point, the underlying mechanism is still unknown. The main purpose of this study is to explore whether and how red culture identity correlates with subjective well-being. In the present study, a large sample of Chinese undergraduates was recruited. In line with the aforementioned studies, we hypothesized that: (1) red cultural identity can significantly positively predict subjective well-being; that is, the stronger the red cultural identity, the higher the subjective well-being; (2) collectivism mediates the effects of red cultural identity on subjective well-being; (3) perspective-taking plays a moderating role in the path from red cultural identity to collectivism; and (4) perspective-taking moderates the psychological effects of collectivism on subjective well-being. The hypothesized model in this study is presented in Figure 1.

## Method

### Participants

A convenience-based cluster sampling of 1,045 Chinese people was recruited from China (683 women). Informed consent was obtained from all participants in the study. The participants were predominantly aged between 18 and 25 years (*n* = 969, 92.73%).

### Measures

#### Subjective Well-Being (SWB)

The Subjective Well-Being Scale (GWBS), as revised by Fazio (1977), was used to measure people’s perceptions of well-being. A localized Chinese version of SWB was applied to the participants of the present study. The localized scale includes 18 items consisting of the 6 factors are: health concerns, energy levels, satisfying interesting life, depressed-cheerful mood, emotional-behavioral control, and relaxed versus tense-anxious (Xin et al., 2021). The items 2, 5, 6, and 7 were scored on a 5-point scale; items 15, 16, 17, and 18 were scored on an 11-point scale; the other items were scored on a 6-point scale. Higher scores signify higher levels of subject well-being. Cronbach’s α was 0.83 for this sample.

#### Red cultural identity (RCI)

This was measured using 20 items consisting of 4 factors: cognitive, emotional, behavioral, and value, which were taken from a previous study (Hu et al., 2014). Each item was scored on a 5-point Likert scale (1 = “does not describe me at all” to 5 = “describes me greatly”), higher scores indicated higher levels of identity in red culture. Cronbach’s alpha was 0.84 for this sample.

#### Collectivism

This was measured using the Confucian Values Scale (Zhang and Jolibert, 2003), which includes 15 items consisting of three factors: collectivism, environmental concern, and face value. Each item was scored on a 5-point Likert scale (1 = “disagree very much” to 5 = “agree very much”), with no reverse scoring questions. The study selected the collectivism dimension for evaluating participants’ collectivist tendencies, which includes 5 items, with higher scores indicating higher levels of collectivism. Cronbach’s alpha was 0.82 for this sample.

#### Perspective-taking (PT)

The Interpersonal Reactivity Index (IRI), developed by Davis (Cliffordson, 2001), was translated into Chinese by Zhan in 1987 (Wang et al., 2020). The localized version includes 22 items consisting of four factors: perspective-taking, empathy concern, fantasy, and personal distress. All items are rated on a five-point Likert scale ranging from 1 (does not describe me at all) to 5 (describes me greatly), with higher scores indicating higher degrees of thinking from the perspective of others, with reverse scoring questions. In this study, one of the perspective-taking dimensions was selected to evaluate perspective-taking ability, which includes 6 items. Cronbach’s alpha was 0.78 for this sample.

#### Demographic variables

The participants’ age groups, gender, and educational level were collected as control factors.

### Statistical analysis

First, scores for all variables were averaged, and the results were normalized. The next step was to perform a Harman single factor test to identify whether there was a common method bias. Then, descriptive statistical analysis of demographic variables was performed using SPSS 21.0, and correlations between collectivism, red culture identity, perspective-taking, and subjective well-being were examined using Pearson correlation analysis. After that, tests for mediating and moderating effects were performed in SPSS using PROCESS 4.0, and the bias-corrected percentile Bootstrap method was used to estimate 95% confidence intervals for the mediating or moderating effects. The demographic variables (i.e., gender, age, educational level) were treated as control factors for all models. Subsequently, a simple effects analysis was performed on the moderating variables at ±1 SD to further investigate the model.

## Results

### Primary analyses

A Harman single factor test was performed to examine whether there was a common method bias after the data collection had been completed. The results found that principal component analysis was used to extract 12 factors; we extracted the factors with eigenvalues ≥ 1, and the first factor accounted for only 21.84% of the total variance, which is over 40% (Richardson et al., 2009; Podsakoff et al., 2012). These results indicate that common method bias was not serious in this study.

### Descriptive statistics

First, Pearson’s correlation analysis was conducted for collectivism, RCI, PT, and SWB (see Table 1). The results demonstrate that collectivism was positively correlated with RCI, PT, and SWB; RCI was positively correlated with PT and SWB; and PT was positively correlated with SWB. Therefore, hypothesis 1 and hypothesis 2 were partially supported.

**Table 1:** Descriptive statistics for all variables.

	1	2	3	4
1. Collectivism	–			
2. RCI	0.57^**^	–		
3. PT	0.48^**^	0.38^**^	–	
4. SWB	0.18^**^	0.18^**^	0.09^**^	–
*M*	3.82	3.76	3.39	4.25
*SD*	0.67	0.69	0.74	0.74

RCI, red cultural identity; PT, perspective-taking; SWB, subjective well-being. *N* = 1,045; **p < 0.01.

### The mediating effect of collectivism

We first examined the direct effect of the RCI on the SWB (see Table 2, Model 1), which illustrated a positive predictive effect of RCI. The demographic variables (i.e., age, gender, educational level) were treated as control factors. To test the mediating effects of collectivism, SPSS model 4 was used to investigate the mediating role of collectivism in the influence of RCI on SWB (Hayes, 2012). The mediating effect was tested using the bootstrap method with a sample of 5,000 to estimate 95% confidence intervals for the mediating or moderating effects.

**Table 2:** Model coefficients for the mediation effects, and the moderation effects.

Consequent						
	Collectivism			SWB		
Antecedents	Coefficient	*SE*	*p*	Coefficient	*SE*	*p*
Model 1						
RCI	–	–	–	0.18	0.04	0.00
Gender	–	–	–	−0.05	0.03	0.10
Age	–	–	–	0.02	0.03	0.61
Education	–	–	–	0.03	0.03	0.37
				ΔR^2^ = 0.03		
				*F* = 9.18, *p* < 0.001		
Model 2						
RCI	0.57	0.03	0.00	0.11	0.04	0.00
Collectivism	–	–	–	0.11	0.04	0.00
Indirect effect						
	–	–	–	Effect	SE	LLCI-ULCI
	–	–	–	0.07	0.02	0.02–0.11
	–	–	–	ΔR^2^ = 0.01		
	–	–	–	*F* = 9.35, *p* < 0.001		
Model 3						
RCI	0.44	0.03	0.00	–	–	–
PT	0.32	0.03	0.00	–	–	–
RCI × PT	−0.07	0.02	0.00	–	–	–
	ΔR^2^ = 0.38			–	–	–
	*F* = 119.79, *p* < 0.001			–	–	–
Collectivism	–	–	–	0.14	0.04	0.00
PT	–	–	–	−0.03	0.04	0.47
Collectivism×PT	–	–	–	0.06	0.03	0.04
	–	–	–	ΔR^2^ = 0.02		
	–	–	–	*F* = 119.79, *p* < 0.001		

RCI, red cultural identity; PT, perspective-taking; SWB, subjective well-being. The effects of control factors were omitted in Model 2 and Model 3.

As shown in Table 2, Model 2, RCI was a significant positive predictor of SWB (*β* = 0.18, *p* < 0.001), and RCI was a significant positive predictor of collectivism (*β* = 0.57, *p* < 0.001). When RCI and collectivism simultaneously predicted SWB, the positive predictive effect of RCI on SWB remained significant (*β* = 0.11, *p* < 0.01), and the positive predictive effect of collectivism on SWB was significant (*β* = 0.11, *p* < 0.01). The results of the mediation analysis indicate that collectivism plays a partially mediating role in the effect of RCI on subjective well-being, with a mediation effect of 0.07 and a mediation effect of 38.89% of the total effect. The 95% Bootstrap confidence interval of the mediation effect was [0.02, 0.11]. The direct effect was 0.11, accounting for 61.11% of the total effect, and the corresponding 95% Bootstrap confidence interval was [0.04, 0.18]. Therefore, hypothesis 3 was supported.

### The moderating effect of perspective-taking (PT)

Since the mediation effect of collectivism on RCI on SWB was found to be significant, the moderating effect of PT on this mediation model was further explored.

**Figure 2:**
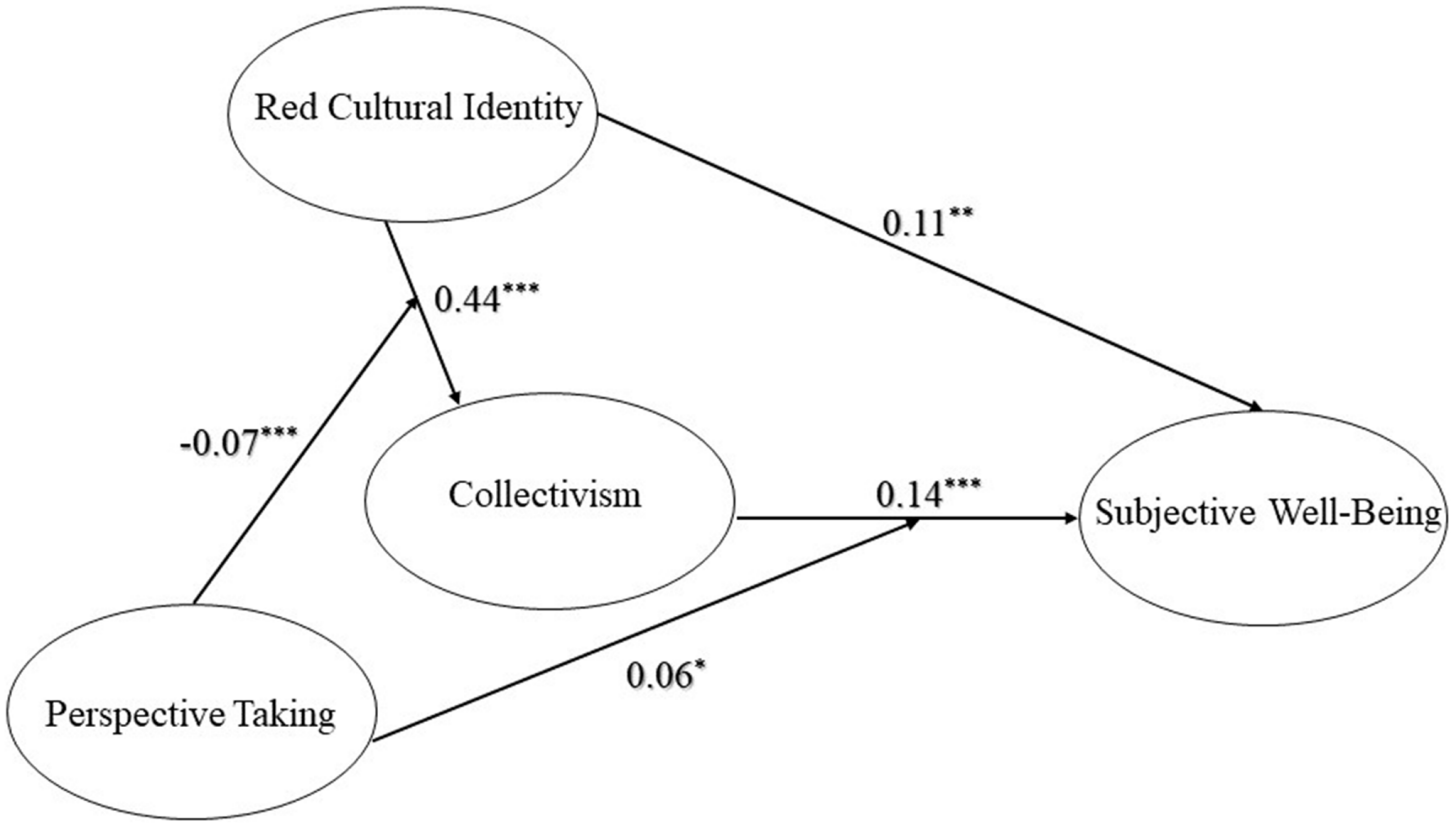
The moderated-mediation model.

As demonstrated in Table 2, Model 3 and Figure 2, regression analysis with gender, age, and educational level as the control variables and collectivism as the outcome variable shows that RCI significantly and positively predicted collectivism (*β* = 0.44, *p* < 0.001) and PT and RCI significantly and negatively predicted collectivism (*β* = −0.07, *p* < 0.01). Besides, while taking SWB as the outcome variable, the result revealed a significant interaction between PT and collectivism that positively predicted SWB (*β* = 0.06, *p* < 0.05).

**Figure 3:**
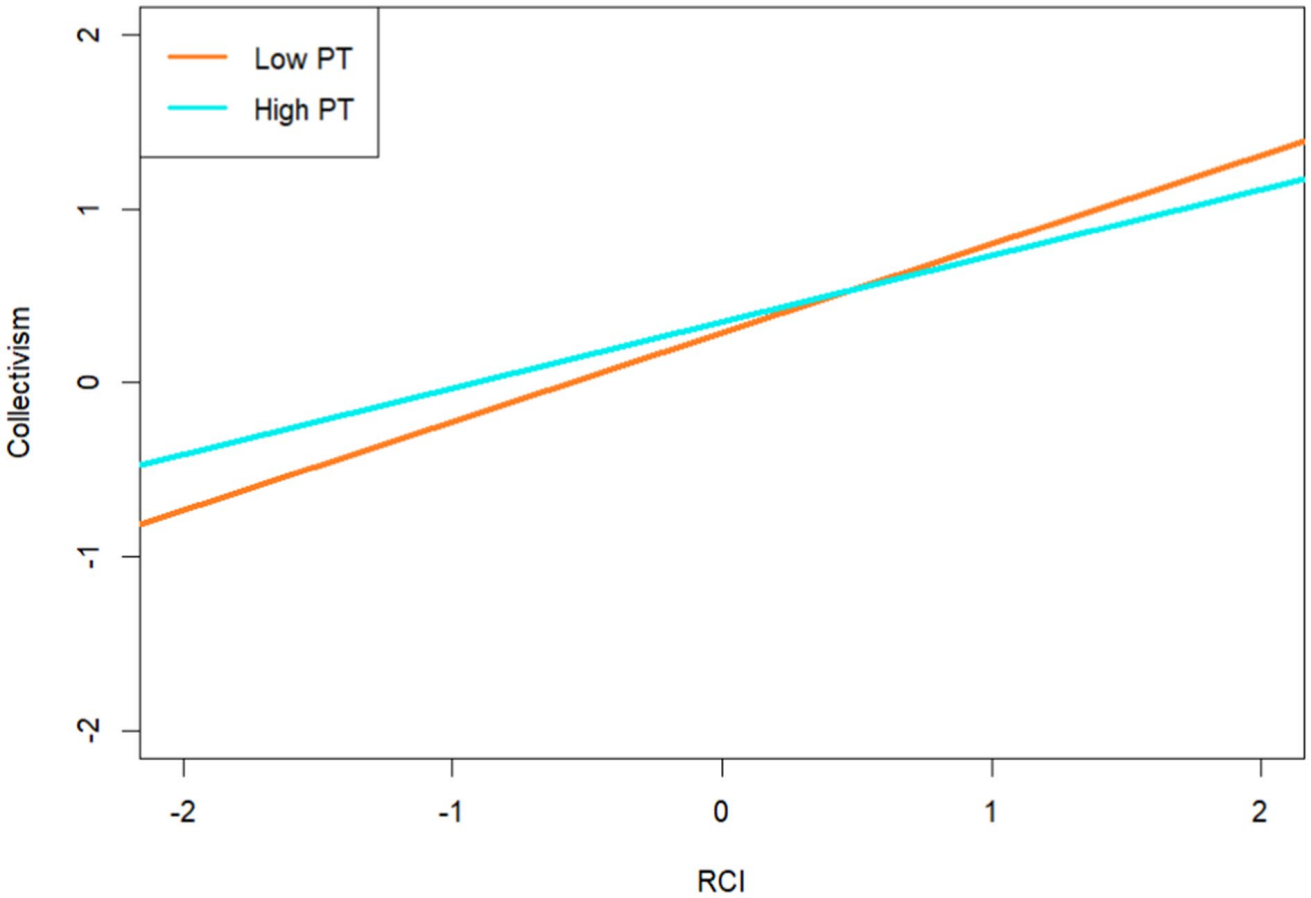
The moderating role of PT between RCI and collectivism.

**Figure 4:**
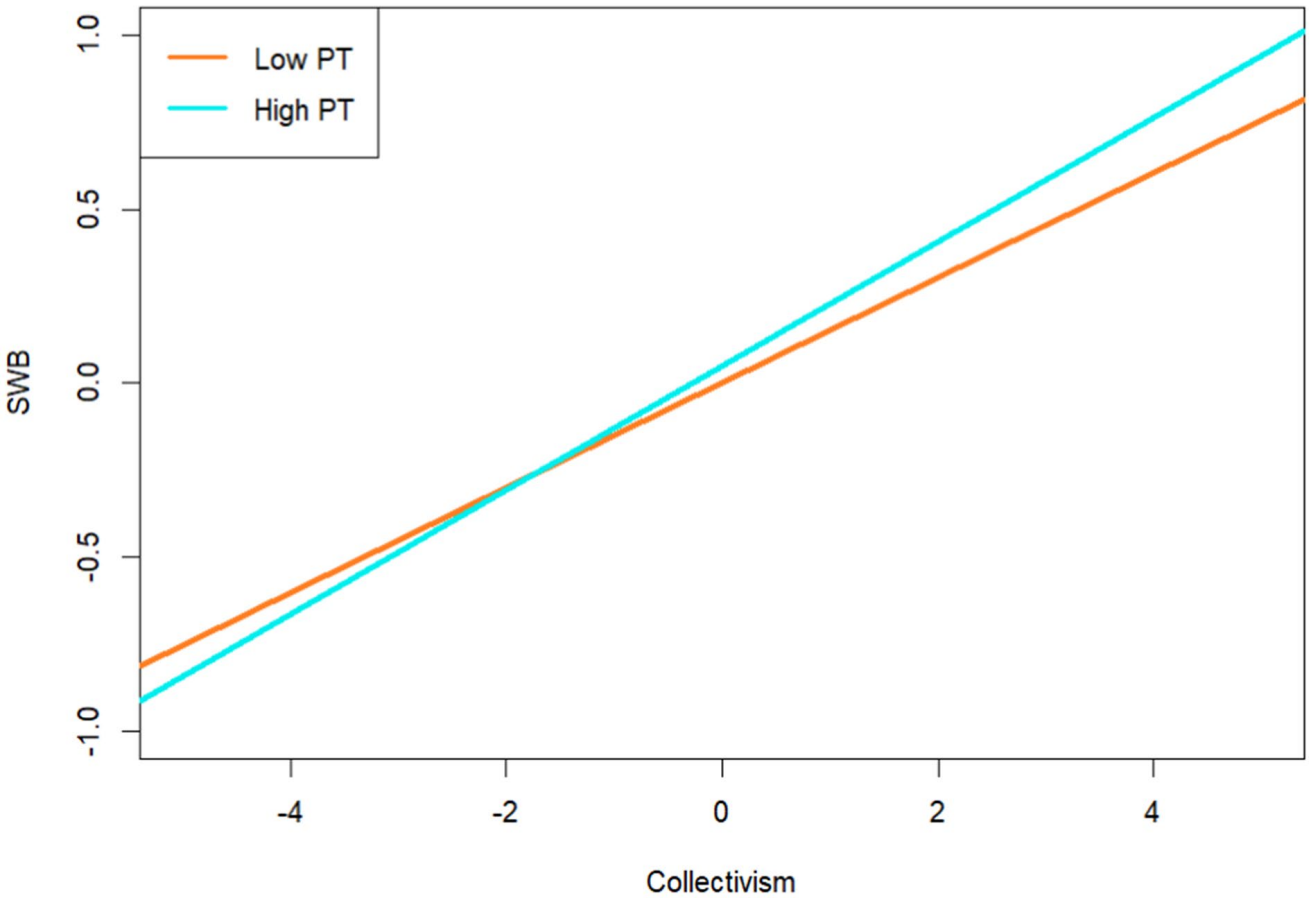
The moderating role of PT between collectivism and SWB.

Furthermore, a simple slope analysis was conducted to better understand the moderating effect of PT. According to Wang et al. (2022), the participants were divided into high and low PT subgroups based on their PT (*M* ± 1*SD*). The findings showed a high subgroup effect value of 0.38 and a confidence interval of [0.31, 0.44], as well as a low subgroup effect value of 0.51 and a confidence interval of [0.45, 0.57]. The predictive effects of RCI on collectivism were calculated when PT was low or high (see Figure 3). In terms of the moderating role between collectivism and SWB, the result demonstrates a high subgroup effect value of 0.20 and a confidence interval of [0.09, 0.31] and a low subgroup effect value of 0.08 and a confidence interval of [−0.01, 0.17] (see Figure 4). The PT was found to moderate the mediating effect of collectivism between red cultural identity and subjective well-being (see Table 3). The conditional indirect effect for low levels of PT was 0.04, with a confidence interval of [−0.01, 0.09]. The conditional indirect effect for PT at the mean level was 0.06, with a confidence interval of [0.03, 0.10]. The conditional indirect effect for high-level PT was 0.08, and the confidence interval was [0.03, 0.12].

**Table 3:** Conditional indirect effects of RCI on SWB.

Mediator	Value of moderator	Effect	Bootstrap SE	Lower level CI	Upper level CI
Low PT (−1SD)	−1	0.04	0.02	−0.01	0.09
Medium PT (mean)	0	0.06	0.02	0.03	0.10
High PT (+1SD)	1	0.08	0.02	0.03	0.12

These results show that the mediating effect of collectivism on SWB is moderated by PT, which confirms hypothesis 4. In particular, the positive predictive effect of collectivism on SWB progressively grew, whereas the positive predictive effect of RCI on collectivism gradually decreased as PT increased. Additionally, only in the high-PT subgroup did PT significantly reduce the association between collectivism and SWB.

## Discussion

The aim of the present study is to explore whether and how red cultural identity is associated with subject well-being by testing a moderated mediating model. These results demonstrate that there was a positive correlation between red cultural identity and subject well-being in which collectivism played a completely mediating role, whereas perspective-taking moderated the relationship between red cultural identity and collectivism and between collectivism and subject well-being. Specifically, those positive correlations were significantly greater for the participants with higher perspective-taking. The present study reveals the distinctive mechanism of how culture affects subjective well-being and provides new perspectives and suggestions for improving subjective well-being, especially in the Chinese cultural context.

In line with general cultural theory, we confirm that red cultural identity can predict subject well-being in a Chinese cultural context. As Social Identity Theory describes, people will assign themselves to the pursuit of their own cultural groups (Ashdown et al., 2011). Individuals’ identity in the culture in which they live denotes the consistency between their opinions and social ideology. Committing to cultural norms reduces the potential for conflicts, which, in turn, leads to a positive personal experience.

From a cross-cultural perspective, an identity with a specific culture is believed to affect the subjective well-being of residents living in the context of that culture. For example, a previous study found that as the duration of residence in Australia increased, Chinese Australian students identified more with and accepted the host national identification (i.e., local Australian individualism). The author stated that cultural identity has a direct positive effect on individuals’ subjective well-being, even when they were not native (Zheng et al., 2004). On the contrary, international students whose cultural backgrounds conflict with the local culture tend to experience more negative feelings when they fail to adapt to the local cultural values (Zheng et al., 2004). The unique effect of local cultural identity is further verified by the World Database of Happiness, which involves 4,500 findings regarding happiness in distinctive countries. For example, immigrants’ levels of happiness were found to increase over time and eventually reach the levels of the native members of the communities in which they live (Veenhoven, 2012). This can be explained by the fact that immigrants gradually identify with the local culture, further indicating a positive relationship between cultural identity and well-being. Schimmack’s study also indicates that the influence of the cognitive component on individual perceptions of well-being is more moderated by culture (Schimmack et al., 2002). Taken together, the subjective well-being of individuals is influenced by the extent to which they identify with the culture they live in.

Importantly, collectivism fully mediates the influence of cultural identity on subjective well-being in the context of Chinese culture. Collectivism emphasizes human interdependence, social embeddedness, and obligation and loyalty to the in-group (e.g., family; Huang et al., 2018) and has long been emphasized as a crucial component of socialist values. As observed in our results, the strong positive correlation between red cultural identity and collectivism implies that the pursuit of collectivism is still advocated by contemporary Chinese culture, which inherited it from revolutionary times. This intrinsic relevance was captured by the full mediation of collectivism between red cultural identity and subjective well-being.

Individuals who live in collectivist societies have a stronger sense of belonging to their own group (Posey et al., 2010). Therefore, red cultural identity gives individuals a sense of belonging. This is consistent with the findings of Dierdorff et al. (2011) that individuals with collectivist orientations might base their identity on group membership as well as value interdependence and the group over themselves, thus promoting the effective functions of the team. From the perspective of the Integrated Mediator–Moderator Mode, the interaction between the environment of red culture and the personality trait of high collectivism can promote life satisfaction, which is a significant part of subjective well-being (Schimmack and Diener, 1997).

Moreover, the present study also verifies that perspective-taking moderates the mediating effect of collectivism on red cultural identity and subjective well-being. In this study, perspective-taking is defined as a cognitive process in which individuals adopt other people’s perspectives in an attempt to understand their preferences, values, and behaviors (Parker and Axtell, 2001). In a red cultural environment, this adoption of others’ perspectives primarily involves identifying with the group in which one is embedded and following others in some red culture-related activities. Interestingly, the positive predictive effect of red cultural identity on collectivism was found to diminish with the increase in perspective-taking. This may be due to the fact that the “face” (also known as “mien-zu”) is more significant in collectivist culture than in individualist culture. In the context of east Asian culture, the “face” is defined as “a reputation achieved through getting on in life, through success and ostentation” (Kim and Nam, 1998), and individuals in a collectivist culture may generate a sense of “being small and worthless” if they lose face, which may lead to negative emotions such as anxiety and fear (Luomala et al., 2015). Individuals who are more concerned about losing face may not participate in such group activities for fear of the unknown consequences of losing face by following the crowd.

It is noteworthy that the predictive effect of collectivism on subjective well-being was significant only for the participants with high perspective-taking. This might be brought on by the differences in prosocial motivation between high-and low-perspective-taking individuals. According to a previous study, people with higher levels of perspective-taking are supposed to have higher levels of prosocial autonomy and stronger motivations for communication, which increases prosocial behavior as a result (Wang et al., 2014). Furthermore, prosocial autonomy motivation allows people to experience a greater sense of well-being (Weinstein and Ryan, 2010), higher life satisfaction (Kwok et al., 2013), more positive emotions, and fewer negative emotions (Gebauer et al., 2008). Therefore, people with higher perspective-taking might be more likely to report higher subjective well-being in a collectivist context. This may be because the effect of perspective-taking on well-being was high enough to allow the role of perspective-taking on subjective well-being to overshadow the role of cultural identity in high-perspective-taking populations.

However, the factors that affect well-being for a particular culture would not play the same role in another culture. For example, perceived social support has been shown to be positively related to overall well-being in collectivist countries (Diener et al., 1995), where increasing individualism would decrease the social support perceived in those countries (Xin and Xin, 2016). Collectivists, who prioritize harmony between the individual and the surrounding environment (Zwolinski, 2019), believe that the collective is the most important thing and focus on achieving common goals (Diener et al., 1995), resulting in a stronger need for social support and emotional attachment. When these factors decrease, individuals’ loneliness increases, which leads to mental health problems and lower levels of well-being. In contrast, in Western countries where people are more concerned with personal liberty, collectivism would not be a critical factor in determining an average individual’s subjective well-being. For example, it was shown that individualism, as opposed to collectivism, shows a significant positive correlation with well-being in Australia, which is generally thought to be an individualistic country (Diener et al., 1995). Individualism is more about equality, liberty, and personal rights and interests, which is contrary to the values of collectivism. Therefore, whether and how a set of values affects subjective well-being depends on the individual’s specific cultural background.

The present study reveals that high perspective-taking leads to higher levels of well-being and positive emotions, which fit with positive psychology. Positive psychology is the science of what is needed for a good life, pursuing the quality of a good life, and devoting oneself to the promotion of well-being (Slade, 2010). The results of the present study also provide suggestions for positive psychology from the perspective of improving subjective well-being.

Despite the merits of the present study, it has several limitations. First, we did not collect the exact age of participants, nor their socio-economic status, eroding the ecological validity of the results. Further study could control for these variables to provide evidence of greater validity. Second, this study was conducted only in China, a typical collectivist society, ignoring the different feelings of individuals in an individualistic society. Cross-cultural studies could be introduced in the subsequent studies, comparing the findings of individualistic and collectivistic societies, and discussing the differences further.

## Conclusion

This study extends the field of cultural identity and well-being research by testing the moderated mediating model in a specific cultural context. The present study clarifies how and when the red cultural identity of Chinese residents is positively correlated with subject well-being. In addition, as perspective-taking represents the ability to engage in social interaction, which is highly valued in collectivist societies, it was found to moderate the mediating role of collectivism in Chinese culture. These results demonstrate that the way cultural identity predicts subjective well-being is highly correlated to specific cultural features, e.g., the opinion of values, which was significant in practice with a cross-cultural background.

## Data availability statement

The raw data supporting the conclusions of this article will be made available by the authors, without undue reservation.

## Ethics statement

The studies involving human participants were reviewed and approved by Institutional Review Board of ethics and human and animal protection committee of the Fujian Normal University. Written informed consent to participate in this study was provided by the participants’ legal guardian/next of kin.

## Author contributions

SZ and RL: conceptualization and methodology. HY: validation, writing—review & editing, and visualization. GL and HY: formal analysis. YH, TH, SL, and JL: investigation. GL and HY: data curation. GL, YH, TH, SL, and JL: writing—original draft preparation. RL: supervision. SZ: project administration and funding acquisition. All authors contributed to the article and approved the submitted version.

## Funding

This work was supported by the Natural Science Foundation of Fujian Province (2022 J05045) and the Innovation Training Program for College Students at Fujian Normal University in 2023 (cxxl-2023386).

## Conflict of interest

The authors declare that the research was conducted in the absence of any commercial or financial relationships that could be construed as a potential conflict of interest.

## Publisher’s note

All claims expressed in this article are solely those of the authors and do not necessarily represent those of their affiliated organizations, or those of the publisher, the editors and the reviewers. Any product that may be evaluated in this article, or claim that may be made by its manufacturer, is not guaranteed or endorsed by the publisher.
